# Conservative Management With a Multimodal Approach of a 12-Week Cervical Ectopic Pregnancy With Fetal Megacystis

**DOI:** 10.7759/cureus.52771

**Published:** 2024-01-23

**Authors:** Daniela Nuti Oprescu, Ana Elena Martiniuc, Monica Mihaela Cirstoiu, Elena Theodora Giubegeanu, Oana Daniela Toader

**Affiliations:** 1 Obstetrics and Gynecology, Institutului Naţional pentru Sănătatea Mamei şi Copilului Alessandrescu Rusescu, Carol Davila University of Medicine and Pharmacy, Bucharest, ROU; 2 Obstetrics and Gynecology, Institutului Naţional pentru Sănătatea Mamei şi Copilului Polizu Bucharest, Bucharest, ROU; 3 Obstetrics and Gynecology, Carol Davila University of Medicine and Pharmacy, Bucharest, ROU; 4 Obstetrics and Gynecology, Institutului Naţional pentru Sănătatea Mamei şi Copilului, Alessandrescu Rusescu, Bucharest, ROU; 5 Institutului Naţional pentru Sănătatea Mamei şi Copilului Alessandrescu Rusescu, Carol Davila University of Medicine and Pharmacy, Bucharest, ROU

**Keywords:** megacystitis, uterine artery embolization, cervical pregnancy, ectopic pregnancy, fertility preservation

## Abstract

Cervical ectopic pregnancy is the rarest kind of ectopic pregnancy, and it is known as the implantation of an embryo into the cervical mucosa. It is commonly associated with complications such as hemorrhage from the cervix and can lead to severe consequences if it is not treated early. For this reason, the treatment for a cervical pregnancy often requires an abdominal hysterectomy. To avoid such radical management, several conservative methods of termination have been used. In this paper, we report a complex management of one of our ectopic cervical cases, which includes embolization of the uterine arteries, treatment with methotrexate and mifepristone, evacuation of the pregnancy followed by local hemostatic sutures and application of a balloon in the cervix. The post-operative period was uneventful. After a three-day postoperative stay, the patient was discharged. The management options employed in the presented case achieved the goal of preserving fertility for our patient. There are no specific guidelines for the treatment of cervical pregnancies in advanced gestational age.

## Introduction

Cervical pregnancy is a rare kind of ectopic gestation and in naturally conceived pregnancies it occurs in about 1% of all ectopic [1]. This type of ectopic pregnancy is associated with a high morbidity rate, with the conventional therapeutic approach being hysterectomy [2]. To preserve fertility, many conservative procedures have been attempted; however, in clinical practice, complex management is frequently used [3]. One of the current therapeutic means is embolization of the uterine artery made angiography: this has been used for the control of bleeding in a lot of gynecological procedures [4]. This procedure has been attempted in the case of cervical pregnancy, with the desire to preserve the uterus. Additional procedures such as local injection of vasopressin, hemostatic sutures, and Foley catheter ballooning were added to reduce bleeding from the cervical area [5-7]. In this report, we present our successful experience using a complex treatment that included embolization of the uterine artery, preoperative medical approach, evacuation of the uterus, hemostatic suture, and a cervically inserted Foley catheter [8].

## Case presentation

A 34-year-old gravida, second gesta, nullipara, referred to our unit from another hospital for a second opinion, at 12 weeks gestation with single-living cervical ectopic pregnancy was admitted to our hospital, after a referral from another center. Her menstruation was regular for the last 13 weeks before the presentation. Her vital signs were within normal limits and findings from physical examination were average except for anemic conjunctiva. A gynecological examination described an enlarged uterus and no adnexal modification. The uterine cervix was hypertrophic and engorged. There was bleeding from the cervical os but in low quantity. 

**Figure 1:**
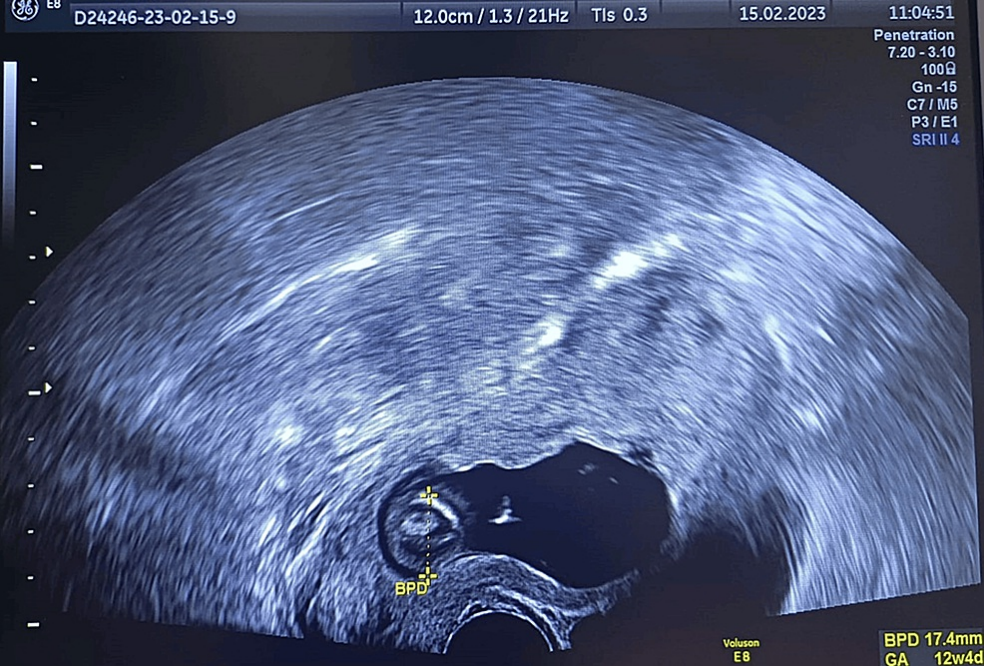
Cervical implantation of a 12-week embryo, with BPD corresponding to 12 weeks and four days

**Figure 2:**
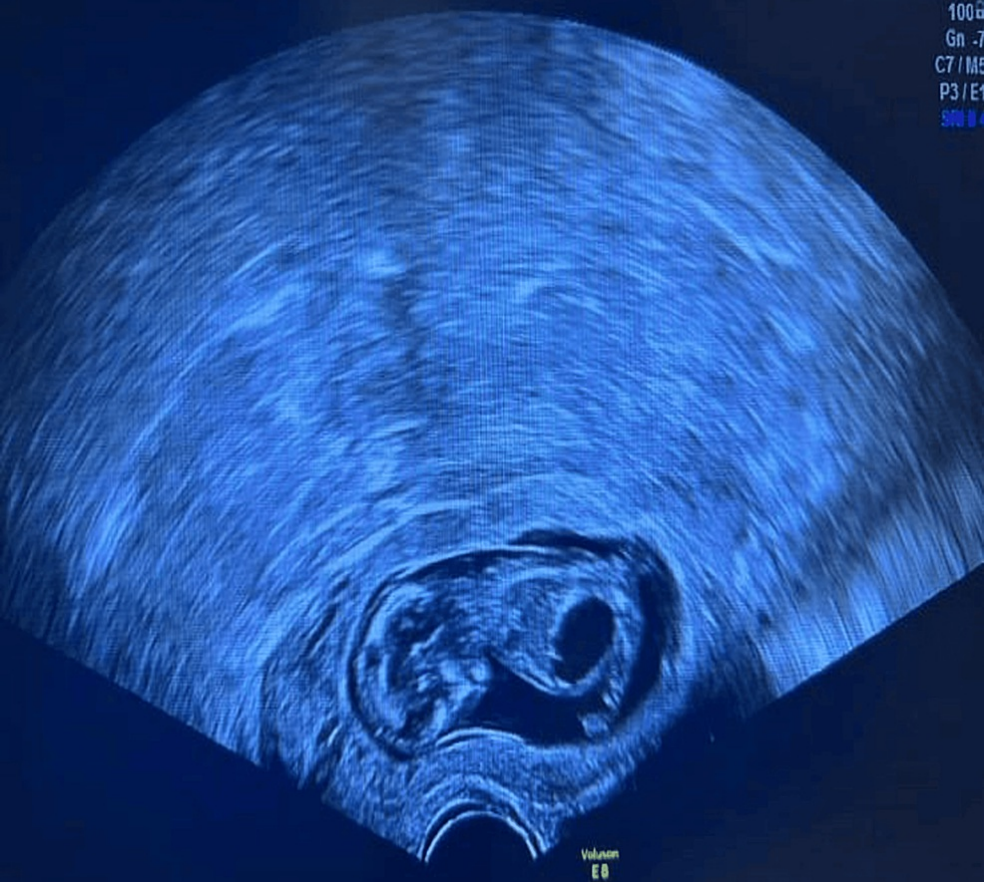
Embryo with megacystis implanted on cervical site

She had a scan performed in another unit that diagnosed a cervical pregnancy. The ultrasound performed in our unit described an empty uterus with no focal masses, the endometrium was thickened with no pseudosac identified. A large gestational sac located in the uterine cervix that contained a single living fetus, crown Rump Length of 53.7 mm that corresponded to gestational age of 12 weeks and four days, with the absence of a sliding sign. The fetus had a longitudinal bladder diameter of 9 mm, consistent with the diagnosis of megacystis. Her menstruations were regular and the last she described that was 13 weeks prior to presentation (Figures 1, 2).

**Video 1:** Cervical pregnancy

 

Laboratory examinations showed a hematocrit of 39.5% and a positive urine - hCG. We did not consider the conventional method D&C followed by tamponade Foley catheter because of the advanced gestational age and because of the high risk of bleeding. The particularity of this case is that the patient was nulliparous, with an expressed desire to preserve fertility.

In an attempt to preserve fertility, we decided to perform a conservative procedure, embolization of uterine arteries before the pregnancy evacuation. We obtained informed consent from the patient, and we discussed with the entire family regarding risks and benefits of the chosen therapeutic protocol.

On admission, the patient was started on intravenous antibiotherapy consisting of cephalosporin. Selective uterine artery and cervicovaginal artery angiograms were acquired under local anesthesia. Uterine arteries showed changes in diameter, they were hypertrophic. Then was performed embolization with absorbable gelatin sponge particles of 1-2 mm diameter and nonionic contrast medium (iopromide (Ultravist 370) until blood flow ceased.

Twenty-four hours after embolization, we decided to administer a single dose of intramuscular methotrexate (100mg), followed by one oral dose of 200mg mifepristone 24 hours later. On the day of the curettage of the cervical pregnancy, the quantitative bHCG was 11.960 UI/L. The procedure was performed under general anesthesia. At the time of the evacuation, the cervical canal measured 2 cm in length and felt like an empty cavity. Blood loss was estimated at 100 mL. After evacuation with manual vacuum aspiration, cervical bleeding continued, which required further intervention with hemostatic suture and insertion of a Foley catheter in the cervix and its balloon inflated with 30 mL normal saline solution for cervical tamponade, that was removed 12h after the procedure.

On the first day after the procedure was done, hematocrit was 31.8% and the pain decreased. The parenteral administration of analgetics and antibiotics continued for about three days and then was switched to oral agents for a further seven days. Ultrasonography performed on the second day showed a normal cervical contour. The patient was discharged on the third post-operative day with vaginal spotting which vanished five days later. Pathologic examination of the specimen obtained by evacuation confirmed the product of conception with a cervical implantation site.

## Discussion

Cervical pregnancy is rare, but it is considered to be a high-risk ectopic situation. Given the rarity of this condition, there is no consensus on the way of treatment: traditionally, cervical pregnancies were treated with hysterectomy, but nowadays conservative treatment is taken into account because of the desire to preserve fertility [9,10].

The differential diagnosis of cervical pregnancy is made by way of ultrasound examination and depends on the stage of gestation. The main diagnosis that has to be excluded is the cervical stage of a miscarriage where the abortus is retained in the cervical canal [11]. Excluding a spontaneous abortion can be difficult, repeated sonography can be diagnostic by confirming sac expulsion or revealing a change in the sac shape and location. The sliding sign described by Jurkovic et al. [6] which describes the gestational sac of an abortion that glides against the cervical canal when applied pressure, may be important in the differentiation of the situations [12]. Another differential diagnosis includes a large Nabothian cyst, in this situation a transvaginal sonography that describes the lack of an echogenic rim and yolk sac establishes the diagnosis [13].

Differentiation of a true cervical pregnancy from an isthmic-cervical pregnancy is very important and is accomplished by the visualization of the closed internal os. The sonographic evaluation must describe local endocervical invasion because the cervical mucosa has little protection against trophoblast invasion and allows penetration into the fibromuscular layer. It is important to evaluate invasion for the diagnosis and monitoring of treatment [6,9,11].

Following diagnosis, treatment is multimodal and personalized in each case. Surgical excision of the trophoblast by curettage or hysterectomy is the classic method. Curettage may be fertility-preserving but has a high hemorrhage risk. Hysterectomy is the safest method, but that involves termination of fertility [8,14].

Reduction of blood supply by cervical cerclage, vaginal ligation of cervical arteries, uterine artery ligation, or angiographic embolization of cervical, uterine, or internal iliac arteries is useful in the preparation of surgical treatment and supports the aim of preserving fertility. Another technique used to reduce blood in the cervical canal is tamponade with a Foley catheter after curettage [15].

Intra-amniotic ultrasound-guided instillation of feticide agents may be used for the limitation of pregnancy, followed by surgical excision. Systemic chemotherapy to terminate the pregnancy, used in single or multiple doses, followed by intensive monitoring may be used as fertility-preserving alternative therapy [16]. Our case was a clinical challenge because it was an advanced gestational pregnancy occurring in a nulliparous patient and the desire for future fertility was expressed.

The particularity of this case is the presence of the fetus with a urinary megabladder. This patient had NIPT at the gestational age of 10 weeks and showed a low risk for trisomy 13, 18, and 21, and no other echographic anomalies were identified. There is no association in literature between fetal anomalies and the place of implantation of the pregnancy and in this case, the patient did not want to continue the genetic investigations after evacuation of the pregnancy.

In an attempt to avoid a major surgical intervention that would adversely affect fertility, we thought of a personalized treatment for our case, which combined minimally invasive procedures and medical therapy, thus we were able to obtain good control in the elimination of the pregnancy and preserve the patient's fertility.

## Conclusions

A personalized combination of different techniques adapted to every situation can be used to preserve fertility in patients with cervical pregnancy. Although the risk of further complications can be higher, personalized medicine with the adaptation of conservative treatments can be a good option for patients who want to preserve their fertility. The multidisciplinary team and the careful follow-up of each surgical procedure led to success, although the experience with cervical pregnancies is small and there is no standard procedure for solving these situations.
